# Multi-colour extrusion fused deposition modelling: a low-cost 3D printing method for anatomical prostate cancer models

**DOI:** 10.1038/s41598-020-67082-7

**Published:** 2020-06-19

**Authors:** Michael Y. Chen, Jacob Skewes, Maria A Woodruff, Prokar Dasgupta, Nicholas J Rukin

**Affiliations:** 1 Redcliffe Hospital, Metro North Hospital Health Service, Queensland, Australia; 2 University of Queensland, School of Medicine, Queensland, Australia; 3 Queensland University of Technology, Science and Engineering Faculty, Queensland, Australia; 4 King’s College London, Guy’s Hospital, London, United Kingdom

**Keywords:** Prostate, Prostate cancer, Surgical oncology

## Abstract

Three-dimensional (3D) printed prostate cancer models are an emerging adjunct for urological surgical planning and patient education, however published methods are costly which limits their translation into clinical practice. Multi-colour extrusion fused deposition modelling (FDM) can be used to create 3D prostate cancer models of a quality comparable to more expensive techniques at a fraction of the cost. Three different 3D printing methods were used to create the same 3D prostate model: FDM, colour jet printing (CJP) and material jetting (MJ), with a calculated cost per model of USD 20, USD 200 and USD 250 respectively. When taking into account the cost, the FDM prostate models are the most preferred 3D printing method by surgeons. This method could be used to manufacture low-cost 3D printed models across other medical disciplines.

## Introduction

Three-dimensional (3D) printing is a technology that allows the creation of complex structures in a layer by layer fashion which was first developed in 1986 but is increasingly being explored in the field of medicine, including urology^1,2^. 3D printed prostate cancer models have shown two potential areas of usefulness: for patient education and surgical planning. Preliminary studies show promising feedback from patients and surgeons^3–6^ with further data on clinical outcomes to follow.

We have previously reviewed the costs associated with urological 3D printed models^1^ and found that the majority of papers do not disclose the costs associated with the models. Only two papers that created 3D prostate models reported costs of USD 500^5^ and USD 317^6^ using the 3D printing technique of material jetting (MJ). In the research setting, manufacturing high quality models is desirable but if these 3D printed models are to become integrated into clinical practice then costs must be considered. It is potentially impractical for healthcare systems or patients to afford this level of expense, particularly when clinical benefits remain unclear^7^.

Therefore, we sought to demonstrate that multi-colour fused deposition modelling (FDM) is a viable low-cost method for creating 3D printed prostate models compared with two other more expensive methods. FDM is the most common and accessible 3D printing method since its patents expired in 2009^8^. FDM printers extrude a thermoplastic filament through a heated nozzle onto a flat platform in programmed layers^8^. FDM printers can be purchased for as little as USD 300 and the thermoplastic filament material for approximately USD 20 per kilogram.

Whilst most low-cost FDM printers can only extrude a single filament and therefore a single colour, newer multi-colour FDM printers can print in multiple colours by either having multiple nozzles or by changing the filament in the nozzle during the print which opens up more possibilities in anatomical modelling^9^.

In this paper, we compare FDM printed prostate models with colour jet printing (CJP) and material jetting (MJ) models and aim to validate this with urologist feedback, whilst also surveying urologists on their preference for model shape, turnaround time for models and overall usefulness. The aim of the study is to determine whether low cost FDM prostate models can be of similar usefulness to more expensive methods in the surgical planning of prostate cancer.

## Materials and Methods

### Prostate model

A de-identified single locally advanced (grade T3, PIRADS 5) prostate cancer MRI scan was used with the patient’s informed written consent. The project received ethics approval from the Royal Brisbane and Women’s Hospital Human Research Ethics Committee (ref: LNR/2019/QRBW/51927) and all methods were carried out in accordance with relevant guidelines and regulations. The MRI was segmented using FDA-approved medical segmentation software Mimics 21.0 (Materialise, Leuven, Belgium) to create a 3D digital model of the prostate, tumour and neurovascular bundles. Segmentation was performed by a urology trainee with 3 months of segmentation and 3D printing experience. This was exported as a stereolithography (STL) file for FDM printing and a coloured Virtual Reality Modelling Language (VRML) file for MJ and CJP printing.

### Printing

**Figure 1:**
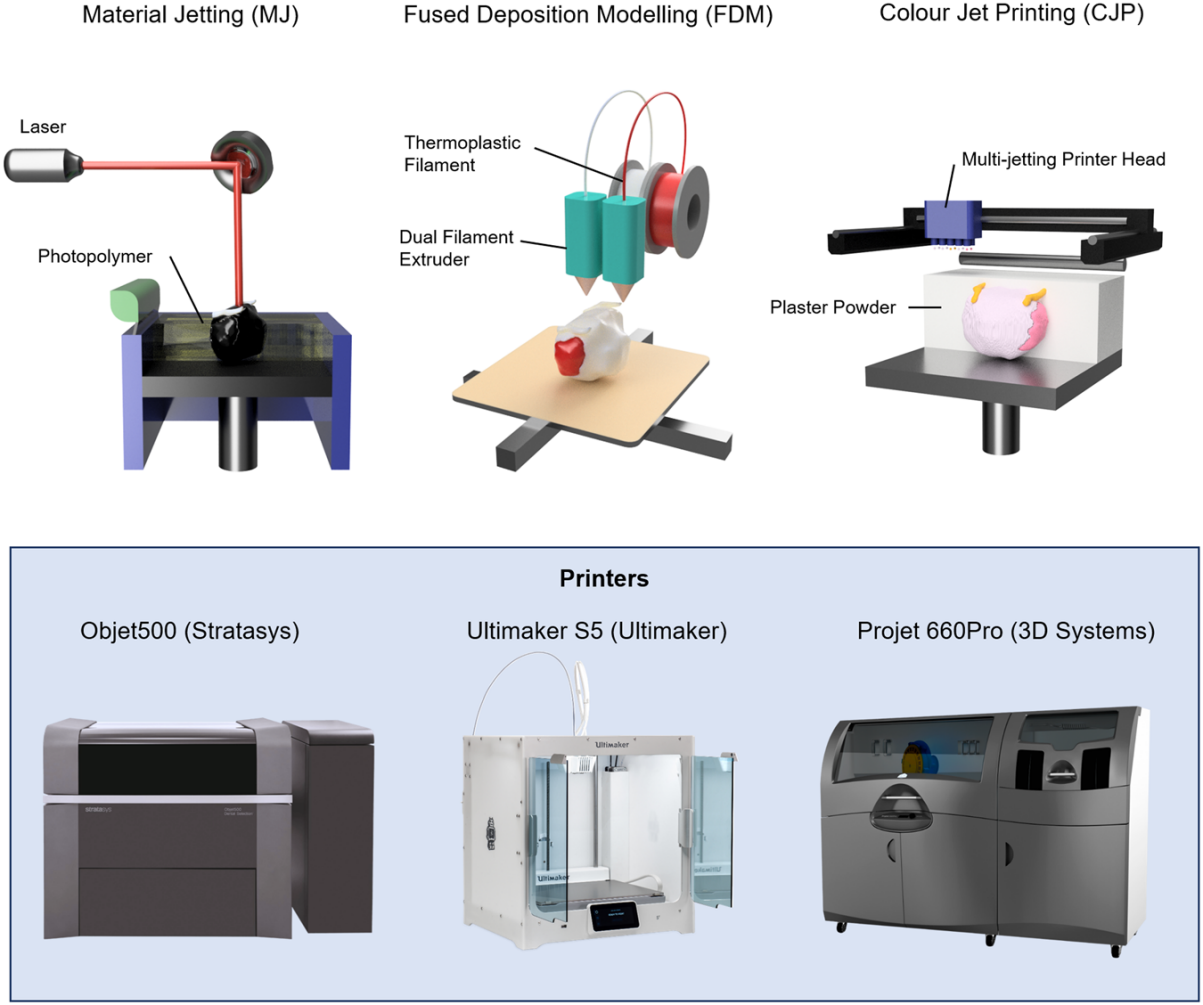
A comparison of the three different 3D printing methods used in this study for creation of anatomical prostate cancer models (top) with their corresponding 3D printer (bottom) and approximate commercial costs for purchase (USD).

Three different 3D printing methods were used for the same prostate model: MJ, FDM and CJP (Fig. 1). For each method, a whole prostate model and a transversely sliced prostate model in two halves were created. Table 1 summarises the three different methods used with associated printer costs and material costs. The estimated cost per model assumes a hospital was to order the models from an external 3D printing company and is based on commercial quotes without shipping costs. These quotes were based on quotes from Australian suppliers and converted to US dollars.

**Table 1:** Comparison of the three different 3D printing methods used to create the prostate cancer models with approximate associated costs.

3D printing Method	3D printer	3D printer cost (USD)	Material	Material cost (USD)	Estimated cost per model (USD)*
Material jetting (MJ)	Objet500 (Stratasys, Rehovot, Israel)	300,000	VeroClear polyjet photopolymer	80	250
Fused deposition modelling (FDM)	Ultimaker S5 (Ultimaker B.V., Geldermalsen, the Netherlands)	10,000	Acrylonitrile butadiene styrene (ABS) filament	1	20
Colour jet printing (CJP)	Projet CJP 660Pro (3D Systems, Rock Hill, SC, USA)	100,000	VisiJet PXL	80	200

*Based on an order of five parts from an external company.

### Survey data collection

**Figure 2:**
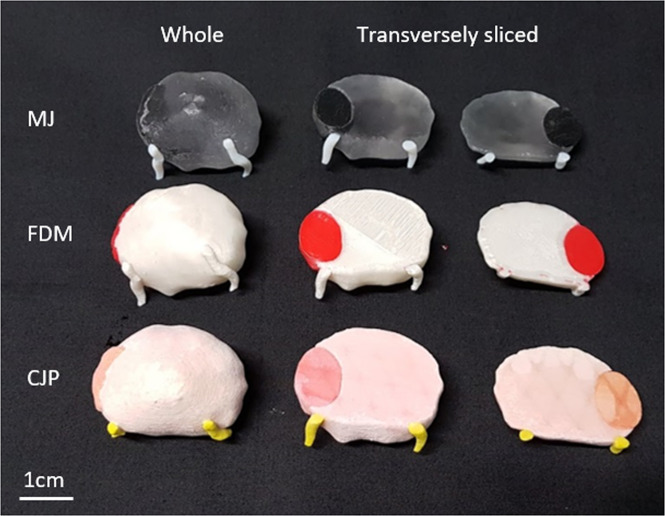
3D printed prostate models manufactured using material jetting (MJ), fused deposition modelling (FDM) and colour jet printing (CJP) for both whole prostate models and transversely sliced models.

The prostate models were shown to 13 consultant urologists and 8 urology trainees at an Australian urology conference as arranged in Fig. 2. Participants had the opportunity to touch and feel the models and were then asked to complete an anonymous written survey. Participants were not told which method was used and were asked to rate their preference for each type of model on a 10 point Likert scale based on their perceived quality. Following this, participants were shown the above estimated costs per model and were again asked to rate their preference for each type of 3D printed model on a 10 point Likert scale, taking into account the costs. Participants were also asked to state their preference for the whole prostate model or the transversely sliced prostate model, their preferred turnaround time to receive a model in public or private practice, as well as their overall impression of the usefulness of 3D printed prostate models in clinical practice. T-test and one-way ANOVA was used to determine statistical significance and performed on Stata/SE version 15.1 (StataCorp LP, College Station, TX, USA).

## Results

Eight urology trainees and thirteen urology consultants participated in the survey. Eleven of the urology consultants had at least five years of experience as consultants. Table 2 shows the ratings from participants for each method of 3D printing blinded and then un-blinded to costs. When blinded to cost, participants preferred the MJ model due to the transparent resin used for the prostate gland but felt they were otherwise interchangeable. However, when shown the cost of each model the FDM model became the most preferred of all three 3D printing methods.

**Table 2:** Ratings from participants on the three different 3D printing methods used before and after being shown the costs associated with each method.

	Cost per model (USD)	Blinded to cost	Unblinded to cost	p value
MJ, mean (SD)	250	8.33 (2.43)	6.70 (0.86)	0.006
FDM, mean (SD)	20	6.86 (1.31)	8.25 (1.77)	0.007
CJP, mean (SD)	200	6.67 (1.62)	5.05 (2.28)	0.01
p value		<0.001	<0.001	

10/13 (77%) consultants preferred the whole prostate models rather than the transversely sliced models, compared to 2/8 (25%) of trainees (p = 0.009). For those surgeons in the public sector, 63% (n = 12) said a 3D model turnaround time of 2–4 weeks was acceptable while 32% (n = 6) wanted a turnaround time of 1–2 weeks. In the private system, 27% (n = 4) wanted models in less than 1 week, 40% (n = 6) selected 1–2 weeks and 33% (n = 5) selected 2–4 weeks. The mean overall rating on the usefulness of 3D printed prostate models was 7.0/10 (SD 1.95). Trainees rated the overall usefulness of prostate models higher but the difference was not statistically significant (7.38 vs 6.75, p = 0.51).

## Discussion

3D printing is an exciting new technology that will transform medicine as the technology continues to improve and becomes cheaper and more accessible over time. Chandak *et al*.^6^ created 3D printed prostate models using MJ on the Objet500, the same MJ printer used in this study, with a stated cost of around USD 317 per model in a pilot study of ten patients with T3 prostate cancer. Their next stages were described as conducting a randomized controlled trial in the future for patients with T3 prostate cancer to determine if these 3D models could improve clinical outcomes, in particular the rate of positive surgical margins.

Other uses for 3D printed prostate models include using them as patient-specific molds to spatially align *in vivo* MRI images with histopathology slides for research purposes^10,11^ as well as for patient education and counselling^3,4^.

These studies into 3D printed models often focus on creating high quality but expensive models. To our knowledge, this is the first study in the literature demonstrating multi-colour extrusion FDM printed anatomical 3D models in medicine. When aware of the costs, most urologists prefer the low-cost FDM printed models and there is a minimal difference in quality even when blinded to cost. New generation FDM printers with multiple extrusion are a promising avenue for the creation of 3D printed patient specific models in the field of medicine. The shape of the 3D prostate models was also explored. In the literature whole prostate models have traditionally been used and this was supported by the consultant surgeons in our study.

Single material FDM printing has been explored in medical uses due to its low cost^12–14^, but in a limited capacity as it was restricted to a single colour in the past. Atalay *et al*.^15^ used FDM printed models of the pelvicalyceal system when planning PCNL surgery as only one colour was needed for this purpose. Chung *et al*.^16^ have similarly aimed to reduce costs in 3D printed abdominal aortic aneurysm (AAA) models, using FDM printers to create models for as cheap as USD 6. We have also published a case of ureteric stent encrustation which was printed on a low-cost FDM printer^17^. However, for most purposes, 3D printed anatomical models will require multiple colours as it is often important to delineate different tissues such as tumours or blood vessels to provide information that can guide surgical planning and education.

Multiple extrusion FDM printing allows for greater flexibility in materials and colours^9^. The Ultimaker S5 used in this study uses two separate nozzles for a maximum of two colours. However, FDM printers are now available that can print up to five materials such as the Prusa MK3S MMU2S (Prusa, Prague, Czech Republic) which is available for approximately USD 2500. This technology will continue to improve in capability and decrease in cost. Therefore, multi-colour FDM printing is a viable low-cost 3D printing alternative for a variety of different anatomies and pathologies in medicine across all specialties and the adoption of this technique and appropriate training of clinical staff is paramount to improve patient outcomes. The low cost also allows for regional centres to perform in-house 3D printing.

Turnaround time is something that must be considered in the workflow of 3D printed models for surgical planning. Surgeons require a short turnaround time to receive these 3D models, particularly in private practice. This is another reason low-cost FDM 3D printing options should be considered for in-house manufacturing of these models in individual institutions to reduce the turnaround time compared to shipping models from a centralized location, often overseas at a high cost. Although FDM may be slower to print than other methods, if the low cost means it can be manufactured in-house, this helps to address some of the other concerns around 3D printed models such as allowing patient data to be kept on site to preserve confidentiality and avoiding the sharing of electronic data, reducing the risk of models going missing or breaking during transport.

The cost of segmentation was not included in this study and in our experience the segmentation of prostate MRI can be challenging. Artificial intelligence (AI) will likely shorten turnaround time by automating the segmentation process in the future and also decrease these costs^18^. The issue of accuracy and goodness-of-fit of segmentation was not covered in this study but is crucial to ensure the safety of any surgical planning done with 3D printed models. T2 windows in prostate MRI are often in 3 mm slices which further complicates segmentation accuracy.

Only a single T3 grade PIRADS-5 prostate cancer model was used in this study. The focus of the study is on these locally invasive T3 grade tumours as these have the highest risk of positive surgical margins, and therefore the most potential clinical benefit from the 3D printed model for planning. However, the results may not be applicable to lower grade intracapsular tumours or multifocal tumours.

Costs in this paper are approximate and are correct at the time of writing (2019) but are expected to change in the future as the technology evolves and becomes more accessible. Commercial quotes may also vary between locations. Labour costs have not been estimated in this study as these can vary significantly based on level of expertise and volume of manufacturing.

## Conclusion

Multi-colour extrusion FDM printing is an exciting low-cost 3D printing approach that can be employed to make 3D prostate models at a fraction of the cost of other 3D printing methods. Although further data is needed on the clinical benefits of these 3D models, these technological developments make these models more cost efficient and accessible for institutions around the world. This low-cost 3D printing method can be employed for other anatomical models across a variety of medical specialties.
